# NKp44 and NKp30 splice variant profiles in decidua and tumor tissues: a comparative viewpoint

**DOI:** 10.18632/oncotarget.12292

**Published:** 2016-09-27

**Authors:** Avishai Shemesh, Aleksandra Kugel, Naama Steiner, Michal Yezersky, Dan Tirosh, Avishay Edri, Omri Teltsh, Benyamin Rosental, Eyal Sheiner, Eitan Rubin, Kerry S. Campbell, Angel Porgador

**Affiliations:** ^1^ The Shraga Segal Department of Microbiology, Immunology and Genetics, Faculty of Health Sciences, Ben-Gurion University of the Negev, Beer Sheva, Israel; ^2^ National Institute for Biotechnology in the Negev, Ben-Gurion University of the Negev, Beer Sheva, Israel; ^3^ Department of Obstetrics and Gynecology, Faculty of Health Sciences, Soroka University Medical Center, Ben-Gurion University of the Negev, Beer Sheva, Israel; ^4^ Institute for Stem Cell Biology and Regenerative Medicine, Stanford University School of Medicine and the Hopkins Marine Station, Stanford, CA, USA; ^5^ Blood Cell Development and Function Program, Fox Chase Cancer Center, Philadelphia, PA, USA

**Keywords:** NKp44, NKp30, tumor, decidua, microenvironment

## Abstract

NKp44 and NKp30 splice variant profiles have been shown to promote diverse cellular functions. Moreover, microenvironment factors such as TGF-β, IL-15 and IL-18 are able to influence both NKp44 and NKp30 splice variant profiles, leading to cytokine-associated profiles. Placenta and cancerous tissues have many similarities; both are immunologically privileged sites and both share immune tolerance mechanisms to support tissue development. Therefore, we studied the profiles of NKp44 and NKp30 splice variants in these states by comparing (i) decidua from pregnancy disorder and healthy gestation and (ii) matched normal and cancer tissue. Decidua samples had high incidence of both NKp44 and NKp30. In cancerous state it was different; while NKp30 expression was evident in most cancerous and matched normal tissues, NKp44 incidence was lower and was mostly associated with the cancerous tissues. A NKp44-1^dominant^ inhibitory profile predominated in healthy pregnancy gestation. Interestingly, the NKp44-2/3 activation profile becomes the leading profile in spontaneous abortions, whereas balanced NKp44 profiles were observed in preeclampsia. In contrast, a clear preference for the NKp30a/b profile was evident in the 1^st^ trimester decidua, yet no significant differences were observed for NKp30 profiles between healthy gestation and spontaneous abortions/preeclampsia. Both cancerous and matched normal tissues manifested balanced NKp30c inhibitory and NKp30a/b activation profiles with a NKp44-1^dominant^ profile. However, a shift in NKp30 profiles between matched normal and cancer tissue was observed in half of the cases. To summarize, NKp44 and NKp30 splice variants profiles are tissue/condition specific and demonstrate similarity between placenta and cancerous tissues.

## INTRODUCTION

NKp44, NKp30, and NKp46 constitute the natural cytotoxicity receptors (NCRs) family that were historically characterized as activating receptors on human NK cells. While a NKp46 homolog is found in mice, there are no murine homologs of NKp44 and NKp30. NKp46 and NKp30 are constitutively expressed on the cell membrane of most NK cells, while NKp44 membrane expression requires stimulation [1, 2]. Both NKp44 and NKp30 mRNA are subject to mRNA splicing events, resulting in three major splice variants per gene (NCR2 and NCR3 respectively) [3].

NKp44 splice variants are named NKp44-1, NKp44-2 and NKp44-3. NKp44-1 possesses an immunoreceptor tyrosine-based inhibitory motif (ITIM) on the receptor cytoplasmic tail, whereas this is missing in both NKp44-2 or -3 variants. Interaction between the ligand PCNA on target cells and NKp44-1 was found to inhibit NK cells functions, but interaction with PCNA with NKp44-2 or -3 did not result in inhibition. Moreover, dominant expression of NKp44-1 over NKp44-2 and NKp44-3 by NK cells is necessary to promote PCNA-mediated inhibition. In acute myeloid leukemia (AML), solitary NKp44-1 expression was associated with poor survival of newly diagnosed patients [4, 5]. In humans, NKp44 protein can be found during pregnancy on decidual NK (dNK) cells, which represent 50-70% of decidual lymphocytes [6, 7].

NKp30 splice variants are named NKp30a, NKp30b and NKp30c. While NKp30a and NKp30b are immune stimulatory isoforms and lead to increased IFN-γ and TNF-α secretion, NKp30c leads to IL-10 secretion and immunosuppression. A NKp30c profile in peripheral blood of gastrointestinal stromal tumor (GIST) patients was linked to poor prognosis [8]. To date, NKp30 splice variants have been studied in cancer, viral infection and pregnancy abortions [9–12].

Placental and cancerous tissues have many similarities. Both trophoblasts and tumor cells exhibit high proliferation rates, which are associated with high levels of PCNA [13, 14]. Both of these cell types can invade normal tissue and promote angiogenesis to establish blood and nutrient supply. In addition, the microenvironment of both tissues maintains immunologic privilege and both share immune tolerance mechanisms to support tissue development [15]. NK cells in placental and tumor tissues share the same phenotype (CD56^bright^, CD16^−/low^) [16, 17]. Moreover, peripheral blood NK cells (CD56^dim^, CD16^+^) cultured in placenta/tumor “like” conditions (hypoxia, TGF-β) have been shown to display a phenotypic shift to resemble dNK/Ti-NK (CD56^bright^, CD16^−^) and to secrete VEGF [18–20]. Several cellular ligands for NKp44 and NKp30 were published [5, 21–25]; trophoblasts, decidua stromal cells, and various cancer cells were shown to express ligands for NKp44 and NKp30 [17]. Recent publications suggest that the placental tissue microenvironment can alter the expression of NKp44 and NKp30 splice variants [12, 26].

In recent years, numerous studies have shown a supportive role for dNK in the formation of the placenta [27, 28]. Moreover, it was shown that tumor infiltrating NK (Ti-NK) cells may play a similar role in tumor development [16, 29]. Therefore, we investigated the splice variant profiles of NKp44 and NKp30 in human clinical samples from tumors and placentas, with the goal of better understanding their biological roles and the similarities/differences between these tissues.

## RESULTS

### NKp44 and NKp30 splice variant profiles in decidual tissue

**Figure 1:**
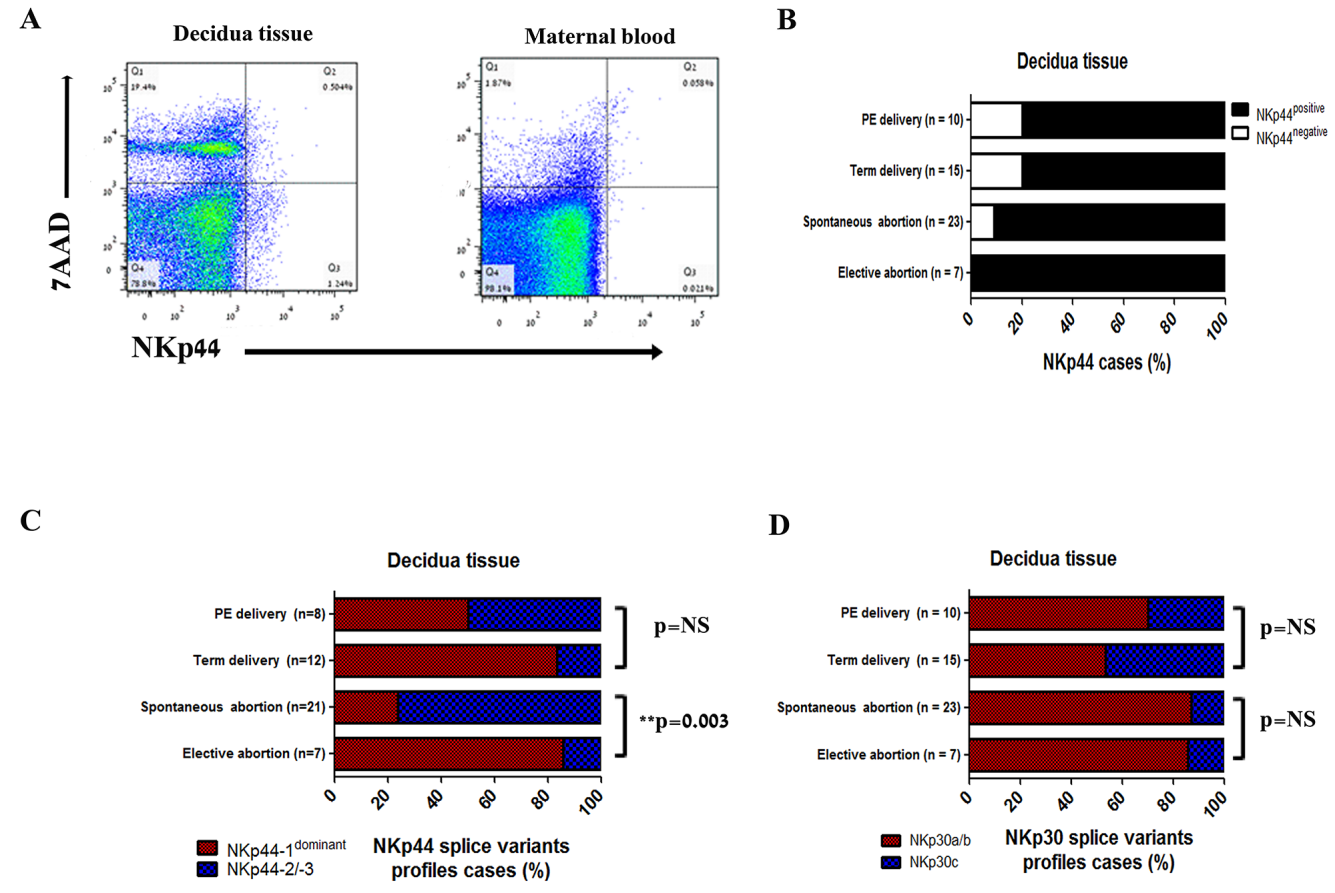
NKp44 and NKp30 splice variants profiles in decidual tissue **A.** Representative flow cytometry analysis for NKp44 protein expression in decidual tissue. Comparison between i) term delivery decidua total cells population, ii) maternal blood PBMC, collected from one birth. Dead cells were excluded by 7AAD. **B.** Percentage of NKp44 mRNA positive (black) and negative (white) cases in i) elective abortions (n= 7), ii) spontaneous abortions (n=23), iii) term delivery (n=15), iv) PE delivery (n=10). NKp44 mRNA was checked by qPCR in placenta samples. **C.** Percentage of NKp44-1^dominant^ profile (NKp44-1 > 66% of NKp44 transcripts – red, inhibitory profile) or NKp44-2/3 profile (NKp44-1 < 66% of NKp44 transcripts – blue, activating profile) in NKp44 positive cases from: i) elective abortions (n= 7), ii) spontaneous abortions (n=21), iii) term delivery (n=12), iv) PE delivery (n=8). NKp44 splice variants were checked by qPCR in placenta samples. Statistical significance was calculated by the Fisher exact test. * p < 0.05, ** p <0.01. **D.** Percentage of NKp30a/b (% NKp30a ≥ % NKp30c – red, activating) and NKp30c (% NKp30a < % NKp30c – blue, inhibitory) cases from: i) elective abortions (n= 7), ii) spontaneous abortions (n=23), iii) term labor (n=15), iv) PE labor (n=10). NKp30 splice variants were checked by qPCR in placenta samples. Statistical significance was calculated by the Fisher exact test. * p < 0.05, ** p <0.01. *NS*: non-significant.

NKp44 protein expression in decidual tissue is well established (Figure 1A) [6, 7]. In order to better understand the NKp44 mRNA variant expression profile in the decidua, we analyzed *decidual* tissue samples from women with pregnancy disorder or healthy gestation by qPCR. Cases were grouped according to the trimester of the pregnancy and clinical condition. We previously reported that dominant expression of NKp44-1 relative to NKp44-2 and NKp44-3 results in suppressed function of NK cells in a PCNA overexpression model system, in which the NKp44-1^dominant^ profile was characterized by NKp44-1 ≥ 66% of total NKp44 transcripts, while the NKp44-2/3 profile was characterized by NKp44-1 < 66% of total NKp44 transcripts [4]. The NKp30 splice variant profiles NKp30a/b and NKp30c were characterized as previously described: the NKp30a/b profile (% NKp30a ≥ % NKp30c; activation) and NKp30c (% NKp30a < % NKp30c; inhibition) [8]

In decidual tissue from elective abortions (1^st^ trimester, n = 7), 100% [7/7] of the cases were positive for NKp44 mRNA. The same trend was seen in decidual tissue from spontaneous abortions (1^st^ trimester, sporadic; n = 16, recurrent; n = 7) with 91.3% [21/23] NKp44 positive cases. In *decidual* tissue from term deliveries and pregnancies complicated by preeclampsia (PE) (n = 15, n = 10 respectively), 80% [12/15], [8/10] of the cases were positive for NKp44 mRNA (Figure 1B).

Recently, Siewiera at el. published that NKp44 and NKp30 splice variant profiles can be altered by the microenvironment through cytokines, such as TGF-β, IL-2, IL-15, and IL-18 [26]. We thereforeexpanded our investigation to include NKp44 and NKp30 splice variant profiles. A NKp44-1^dominant^ profile was found in 6/7 (85.71%) of the elective abortion cases (Figure 4C). In contrast, the NKp44 splice variants profile in spontaneous abortions differed significantly from elective abortions. NKp44-2/3 was the dominant profile found in 16/21 (76.19%) of spontaneous abortions cases (Figure 1C). No difference was seen between the decidual profiles of NKp44 splice variants in sporadic and recurrent abortions (11/15; 73.33%, 5/6; 83.33% respectively; data not shown). We had previously published that NKp30a expression is increased in decidual samples from sporadic abortions relative to elective abortions. However, no difference was observed in the decidual profile of NKp30 splice variants in these two groups (Figure 1D).

The decidual profile of NKp44 splice variants in term delivery did not differ from the profile of elective abortions; 10/12 (83.33%) of cases showed a NKp44-1^dominant^ profile (Figure 1C). However, the decidual profile of NKp30 splice variants did not favor a NKp30a/b profile with 8/15 (53.33%) of cases exhibiting a NKp30a/b profile, while 7/15 (46.66%) showed a NKp30c profile (Figure 1D). Furthermore, in decidual tissue from PE labor, a clinical condition that is not associated with abortion, but is characterized as impaired placental structure of the spiral arteries, the profile of NKp44 splice variants was not favored [NKp44-1^dominant^ or NKp44-2/3 (50%/50%)], while the profile of NKp30 splice variants showed higher incidence of a NKp30a/b profile, as was seen in 1^st^ trimester decidual tissue.

### NKp44 and NKp30 mRNA in human tumor tissue

RNA-seq data of human cancer biopsies is available on The Cancer Genome Atlas (TCGA). First, we clustered the different tumor types by the organ of origin: breast (BRCA), lung (LUAD, LUSC), cervical/uterine (CESC, UCEC, UCS), Kidney (KIRC, KIRP, KIRH), Gastrointestinal tract organs (ESCA, STAD, COAD, READ), and Gastrointestinal (GI) tract accessory organs (LIHC, PAAD, CHOL). Each cluster represents a unique immunological niche. We then analyzed the primary solid tumor (PST) tissue samples with or without matched solid tissue normal (STN) for NKp44 and NKp30 mRNA (Table 1: TCGA samples used in this study).

**Table 1:** TCGA samples used in this study

*Cancer Cluster*	*Cancer Name*	*TCGA acronym*	*N*_PST_	*N*_STN_
Breast	Breast Cancer	BRCA	1095	113
Lung	NSCLC, Adenocarcinoma	LUAD	515	58
	NSCLC, Adenocarcinoma	LUSC	216	21
			731	79
Cervical/Uterine	Cervical Squamous Cell Carcinoma & endocervical adenocarcinoma	CESC	304	3
	Uterine Corpus Endometrial	UCEC	544	22
	Uterine Carcinosarcoma	UCS	57	0
			905	25
Kidney	Kidney Chromophobe	KICH	66	25
	Renal clear cell carcinoma	KIRC	533	72
	Renal papillary cell carcinoma	KIRP	290	32
			889	129
Gastrointestinal tract organs	Colon adenocarcinoma	COAD	440	39
	Esophageal carcinoma	ESCA	183	11
	Rectum adenocarcinoma	READ	166	10
	Stomach adenocarcinoma	STAD	415	32
			1204	92
Gastrointestinal (GI) tract accessory organs	Cholangiocarcinoma	CHOL	36	9
	Liver hepatocellular carcinoma	LIHC	371	50
	Pancreatic adenocarcinoma	PAAD	178	4
			585	63

*Cancer clusters, names and number (N) of PST and STN cases from TCGA.

**Figure 2:**
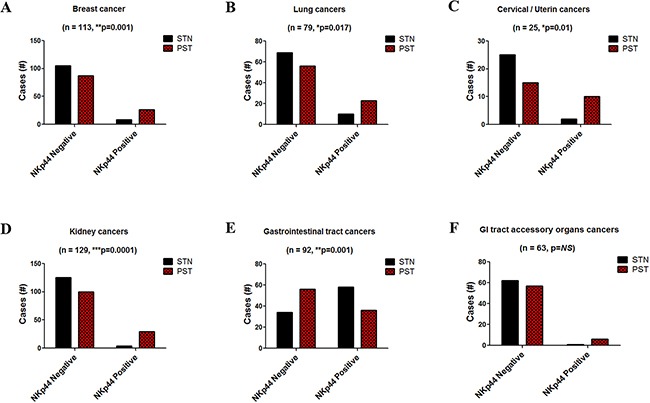
NKp44 mRNA incidence in cancer cases RNAseq gene expression data from the TCGA was analyzed for the incidence of NKp44 mRNA positive cases in primary solid tumor (PST- red) samples and paired solid tissue normal (STN - black) control samples. Cancer types were clustered by organ. **A.** Breast (BRCA). **B.** Lung (LUAD, LUSC). **C.** Cervical/Uterine (CESC, UCEC). **D.** Kidney (KIRC, KIRP, KIRH) **E.** Gastrointestinal tract organs (ESCA, STAD, COAD, READ) **F.** Gastrointestinal (GI) tract accessory organs (LIHC, PAAD, CHOL). Statistical significance was calculated by the Fisher exact test of clustered STN cases (NKp44 negative and positive) vs. PST cases (NKp44 negative and positive). * p < 0.05, ** p <0.01, *NS*: non-significant. Normalized RNAseq data (RSEM) was obtained from the TCGA (IlluminaHiSeq_RNASeqV2.Level_3).

As previously mentioned, NKp44 expression requires stimulation. Accordingly, we studied the incidence of NKp44 mRNA in PST relative to matched STN samples. Consistent with previous publications [30–32], the incidence of NKp44 mRNA was significantly higher in PST compared to STN samples in breast (n=113), lung (n=79), cervical/uterine (n=25), and kidney clusters (Figure 2A,B,C,D). In the gastrointestinal tract organs cluster (n=92), high incidence of NKp44 mRNA were found in both PST and STN samples, which is consist with previous publications of NKp44+ cells found in the GI tract of healthy individuals [33, 34]. However, incidence of NKp44 mRNA was significantly lower in PST compared to STN samples, possibly because of an increase in the number of tumor cells (Figure 2E). In the gastrointestinal (GI) tract accessory organs cluster (n=63), no significant change was detected between PST and matched STN samples (Figure 2F). With respect to NKp30, 99% of PST and STN samples were positive for NKp30 mRNA. However, NKp30 mRNA expression did not increase significantly in PST compared to STN matched samples, with exception of the kidney cancers cluster. Moreover, NKp30 mRNA expression was significantly decreased in PST samples of lung and gastrointestinal (GI) tract accessory organs clusters (RSEM normalization; Supplementary Figure S1) [35], which concurs with earlier publications [32, 36].

Increased incidence of NKp44 mRNA expression could be a result of higher numbers of NK cells infiltrating the tumor tissue. However, when comparing the expression of NKp46 mRNA between PST and STN samples for each cluster, no significant changes were observed with the exception of the kidney tumor cluster, which showed significantly higher expression levels of NKp46 mRNA (RSEM normalization; Supplementary Figure S2) [35].

In order to validate our results from the TCGA RNA-seq data, we studied the incidence of NKp44 mRNA and the expression of NKp30 and NKp46 in lung tumor biopsies with matched normal tissue (n =18) by qPCR. Consistent with the TCGA data, NKp44 mRNA incidence was significantly higher in the tumor tissue compared with the matched normal tissue. However, the number of tumor cases positive for NKp44 mRNA was higher (Supplementary Figure S3A). Moreover, expression of both NKp30 and NKp46 mRNA was reduced in tumor tissue relative to normal tissue (Supplementary Figure S3B,C). Hence, higher incidence of NKp44 mRNA is associated with tumor tissue.

### NKp44 splice variants profiles in tumor tissue

**Figure 3:**
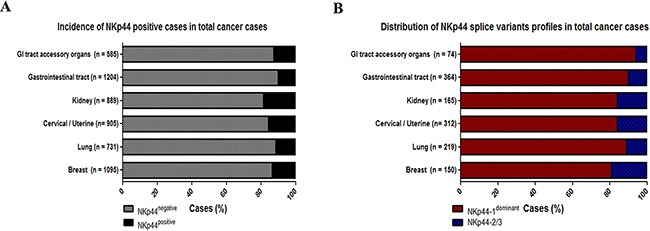
NKp44 splice variants profiles in cancer tissues RNAseq isoforms expression data from the TCGA was analyzed for the incidence of NKp44 mRNA positive cases and NKp44 splice variants profiles in total primary solid tumor cases. **A.** Percentage of NKp44 positive (black) and NKp44 mRNA negative (white) cases in total PST cases per cancerous cluster. **B.** Percentage of NKp44-1^dominant^ profile (NKp44-1 > 66% of NKp44 transcripts – red, inhibitory profile) and NKp44-2/3 profile (NKp44-1 < 66% of NKp44 transcripts – blue, activating profile) in NKp44 positive total PST cases. Normalized RNAseq data (RSEM) was obtained from the TCGA (IlluminaHiSeq_RNASeqV2.Level_3).

NKp44 and NKp30 splice variant profiles were previously shown to promote diverse effector functions, but were never fully characterized in human tumor tissues. Therefore, we investigated the splice variant profiles of NKp44 and NKp30 in tumor tissues by analyzing the total PST samples of the relevant human cancer biopsies available on TCGA (Table 1: TCGA samples used in this study). NKp44 mRNA incidence ranged from 10 to 20% positive cases in the various cancer-type clusters (Figure 3A). We then analyzed the NKp44 positive cases in each cluster of cancer types for the NKp44 splice variant profiles by counting the number of samples in each cluster of cancer with NKp44-1^dominant^ or NKp44-2/3 profiles to create cancerous cluster profiles of NKp44 splice variants (Figure 3B). In all clusters of cancer, the NKp44-1^dominant^ profile was the most abundant, ranging from 80%-100% of the cases. The same trend in NKp44 splice variant profile distribution was observed in STN samples (with respect to the lower incidence of NKp44 mRNA), which may indicate an effect of the tumor microenvironment on the surrounding normal tissue (data not shown, Supplementary Figure S3D).

### NKp30 splice variant profiles in tumor tissue

**Figure 4:**
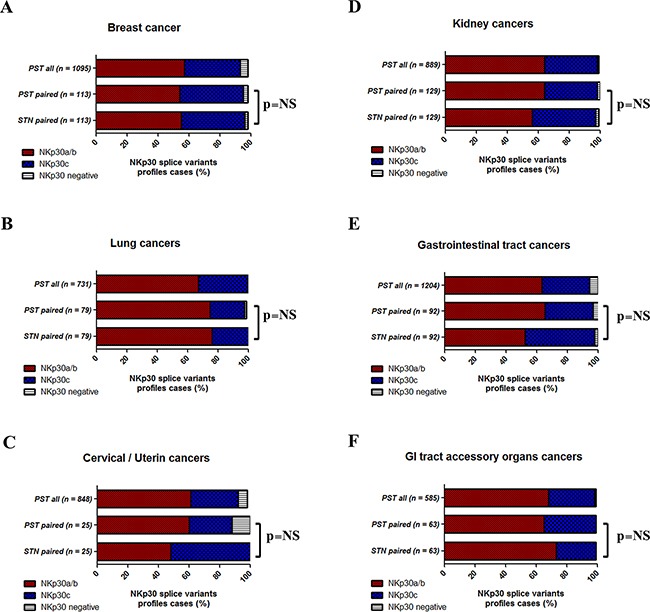
NKp30 splice variants profiles in cancer tissues RNAseq isoforms expression data from the TCGA was analyzed for the incidence of NKp30 splice variants profiles in i) total PST cases, ii) PST cases with matched STN, iii) STN matched cases. Percentage of NKp30a/b (% NKp30a ≥ % NKp30c – red, activating) and NKp30c (% NKp30a < % NKp30c – blue, inhibitory) and NKp30 negative cases (white). **A.** Breast, **B.** Lung, **C.** Cervical/Uterine, **D.** Kidney, **E.** Gastrointestinal tract organs, **F.** Gastrointestinal (GI) tract accessory organs. Statistical significance was calculated by Fisher exact test of clustered STN cases vs. PST matched cases or total PST cases (NKp30a/b and NKp30c). * p < 0.05, ** p <0.01, *NS*: non-significant. Normalized RNAseq data (RSEM) was obtained from the TCGA (IlluminaHiSeq_RNASeqV2.Level_3). PST cases with no matched STN cases were excluded.

In the tumor tissues and paired normal tissues, 99% of cases were positive for NKp30. We then expanded our research to NKp30 splice variant profiles by determining the incidence of NKp30a/b and NKp30c (Figure 3), and created NKp30 splice variant profiles for each cancer cluster. We compared the distribution of NKp30 splice variant profiles between 1) STN samples, 2) matched PST samples, and 3) total PST samples for each cluster of cancers. The distribution of NKp30 splice variant profiles in all clusters was about 60% NKp30a/b cases and 40% NKp30c cases. However, no significant change was detected between the PST and paired STN sample groups. Hence, tumor profiling of NKp30 splice variants did not differ from normal tissue (Figure 3, Supplementary Figure S3.E). We then compared the NKp30 splice variants profile between STN and matched PST samples in each patient for each tumor type and grouped them into identical (STN = PST) or non-identical (STN ≠ PST) NKp30 splice variant profile cases (Figure 4A, S3.F). On average, 50% of STN and PST matched cases were non-identical for NKp30 splice variant profile. Accordingly, we tried to classify the NKp30 splice variants profile shift (NKp30a/b → NKp30c or NKp30c → NKp30a/b) between STN to PST. However, in most tumors, the NKp30 splice variant profile did not favor NKp30a/b → NKp30c or NKp30c → NKp30a/b transitions. Tumor cells and the normal cells they derived from have a profound genetic distance; also, the cancer microenvironment is distant from the microenvironment in the normal tissue in which the cancer originated. The shift in immune status between microenvironment of cancer and microenvironment of matched normal tissue, including e.g. the shift in NKp30 splice variants that we observed, could serve as a biomarker for the cancer-associated virulence mediated by the cancer immunome.

## DISCUSSION

NKp30 and NKp44 splice variant profiles were previously shown to influence the outcome of NK cell function *in vitro* and were associated with poor survival of GIST and AML patients, respectively [4, 8]. With respect to NKp44, we and others have published that NKp44 can mediate inhibition of NK cell and pDC functions [5, 37, 38]. Moreover, the balance between NKp44^ITIM+^ (NKp44-1) and NKp44^ITIM-^ (NKp44-2 or -3) splice variant expression can affect inhibition mediated by NKp44 upon PCNA recognition and can result in poor formation of stable lytic immune synapses by NK cells [4]. Recently, Siewiera et al. published that both NKp30 and NKp44 splice variant profiles can be influenced by factors in the decidual microenvironment, which it is in line with our previous publication. In vitro, peripheral blood NK (pNK) cells cultured in decidua “like” conditions acquired a decidual NK (dNK) cell profile of NKp30 and NKp44 splice variants and became less cytotoxic [26].

In this study, we aimed to investigate the splice variant profiles of NKp44 and NKp30 in decidual and tumor tissues, to our knowledge for the first time, using minimally invasive methods without the involvement of NK cell isolation and culturing. Our results indicate the existence of specific NKp44 and NKp30 splice variant profiles with similarities between placenta and cancerous tissues.

Term delivery decidual tissue and tumor relative to matched normal tissues elicited balanced NKp30 splice variant profiles and NKp44-1^dominant^ inhibitory profiles (Figure 1C, 1D, 3B, 4). Both tissues are chronically tolerated by the immune system. The tumor microenvironment is similar to the placental microenvironment in establishing blood and nutrient supply, and both microenvironments actively modulate the immune response [15]. As in the placenta, the tumor microenvironment also exhibits hypoxia and is associated with TGF-β secretion, factors that reduce the cytotoxic activity of tumor infiltrating NK (Ti-NK) cells and encourage their ability to secrete VEGF [19, 20]. Additionally, Ti-NK cells acquire a decidua-like phenotype in lung, melanoma, breast and colon tumor tissues [16, 30]. All together, these results support the impression that term delivery decidua and tumor microenvironment factors influence NK cells and NKp44 and NKp30 splice variant expression profiles in order to achieve tolerance.

**Figure 5:**
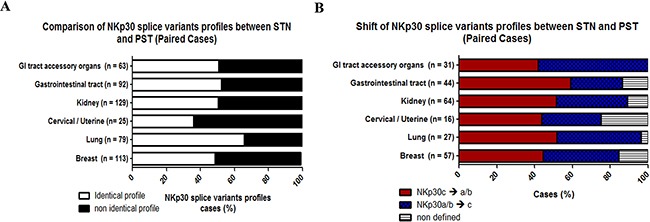
NKp30 splice variants shift between STN to PST NKp30 splice variants profiles were compared between STN and matched PST samples in i) Breast, ii) Lung, iii) Cervical/Uterine, iv) Kidney, v) Gastrointestinal tract organs, vi) Gastrointestinal (GI) tract accessory organs cancerous clusters. **A.** Percentage of Identical profile (STN = PST, white) and non-identical profile (STN ≠ PST, black). **B.** Percentage of cases that exhibit a NKp30c → a/b (red) or NKp30a/b → c (blue) shift. Matched cases that were negative for NKp30 mRNA in PST or STN tissue were classified as non-defined (white). Normalized data (RSEM) was obtained from the TCGA (IlluminaHiSeq_RNASeqV2.Level_3).

As Siewiera *et al*. observed *in vitro* [26], by comparing profiles between paired normal and tumor samples; we were able to show *in vivo* that the NKp30 splice variant profile can be subject to changes at the tissue level (Figure 5). However, no favorable shift in NKp30 splice variant profile was observed, which could indicate a balance between the “need” for immune activation to reject the tumor and the “need” for tumor initiated immune suppression. The NKp30a/b profile promotes secretion of inflammatory IFN-γ and TNF-α, while the NKp30c profile is linked to immunosuppressive IL-10 production [8]. Moreover, the NKp44-1 profile leads to decreased secretion of IFN-γ [4], however the full effect of the NKp44-1 profile on secretion of cytokines, such as TNF-α /IL-10/IL-4, and angiogenesis factors, such as VEGF, is still poorly characterized. The balance between type-1 and type-2 cytokines is essential to the success of pregnancy and the establishment of immune-tolerance towards the fetus [39]. A balance of type-1 and type-2 cytokines was linked to poor survival in epithelial ovarian cancer [40]. Moreover, cytokines in the tumor microenvironment have been shown to be essential mediators of tumor growth, immune tolerance, angiogenesis and tissue remodeling [41, 42].

In line with our results, NKp44 mRNA and protein expression were increased in various tumor tissues [30, 31]. Decidua and tumor tissues are both characterized by high levels of PCNA expression [13, 14]. Indeed we showed PCNA over expression in cancer for the current study by comparing PCNA expression in matched normal and cancer tissue (Supplementary Table S1, TCGA data). Tumor and term delivery decidual tissues showed NKp44-1^dominant^ inhibitory profiles. We and others published that PCNA can be used by tumor cells to suppress NK cell functions [4, 5, 37, 43]. Therefore, overexpression of PCNA in tumor tissue associates with bias towards NKp44-1^dominant^ inhibitory profile. Yet, PCNA overexpression is imperative for cancer virulence in broad spectrum of functions; thus, PCNA-mediated immune suppression is probably not the leading drive for its cancer-associated overexpression. As with cancer cells, trophoblasts also exhibit high proliferation rates with high levels of PCNA protein [13]. It is possible that the mechanism by which cancer cells suppress NK cell lytic function through NKp44 evolved through the “need” of the trophoblasts to form the placenta and support the fetus for a long duration.

In contrast to term delivery and elective abortions, decidual tissue from spontaneous abortions showed an activating splice variant profile of NKp30 (Figure 1D). Only the NKp44 splice variant profile was different in decidual tissue between the elective and spontaneous abortion groups (Figure 1C). We had previously reported that expression of NKp30a is increased in the placental tissue of women with spontaneous and recurrent abortions and that the correlation between decidua-related cytokines and NKp30 is lost [12]. NKp30 ligand is express on decidual stromal cells, while NKp44 ligand was found on trophoblasts, raising the question of how decidual NK cells interact with stromal cells and trophoblasts during abortions and its effect on the decidual cytokine profile [17]. NK cells can secret TNF-α, a pro-inflammatory cytokine that can induce apoptosis of trophoblasts and cancer cells, and affect the remodeling of spiral arteries [27, 39]. Moreover, blocking TNF-α with anti TNF-α mAb in woman with RPL was shown to increase the live birth rate. It may be that the factors that promote the shift in NKp30 and NKp44 splice variant profiles can be used to promote successful tolerance toward the fetus in women with first trimester abortions or tumor rejection in cancer patients.

## MATERIALS AND METHODS

### TCGA data

Data was downloaded from The Cancer Genome Atlas (TCGA Data Portal; https://tcga-data.nci.nih.gov/tcga/). Data was extracted from normalized (RSEM) RNA-Seq-V2 results (i.e. “rsem.gene.normalized_results” and “rsem.isoforms.normalized_results” file types [35]). Further data preprocessing and mentioned analyses were performed in the R statistical environment (http://www.r-project.org). Sample type was identified through the TCGA sample and aliquot barcode, using the 5^th^ section of the barcode considering dashes as section separators. For example, in the barcode (TCGA-AB-2842-03A- 01T-0734-13) the underlined section was taken to indicate sample type. Only sample types 01 = Primary Solid Tumor (PST) and 11 = Solid Tissue Normal (STN) were considered. Analytic: T = total RNA. Percentage of NKp44/NKp30 splice variants from total NKp44/NKp30 mRNA was calculated by dividing the TCGA expression levels of individual splice variants by the total NKp44/NKp30 expression. Table 1. TCGA samples used in this study.

### Study design and population

A single center prospective case-control study was conducted in the Department of Obstetrics and Gynecology at the Soroka University Medical Center, Beer Sheva, Israel in collaboration with the Clinical Biochemistry Laboratory at Ben-Gurion University. The study was approved by the ethical institutional review board (in accordance with the Helsinki declaration).

1^st^ trimester decidual samples: The study population was comprised of women who presented to the Soroka Medical Center for dilation and curettage (D&C) during the first trimester of pregnancy (<14 weeks of gestation), and consented to participate in the study as was described elsewhere [12]. Women with known uterine anomalies, multiple gestations, and women undergoing elective curettage who had a history of ≥ 2 spontaneous pregnancy losses were excluded from the study. The placental tissue samples were: elective n=7, sporadic n=16, recurrent n = 7 (spontaneous abortions: n = 23). **Approval number: 0267-12-SOR.**

3rd trimester decidual samples: The study population was comprised of women presenting to the Soroka Medical Center during the third trimester of pregnancy (>30 weeks of gestation), who consented to participate in the study. Placenta samples were collected from patients with preeclampsia (n=10), and from patients following normal term deliveries (n=15) after birth. Tissue samples were taken from the surface of the maternal side of the placenta. **Approval number: 0194-11-SOR.**

Frozen samples of lung cancer tumors and surrounding normal lung tissue from 18 patients were obtained in de-identified form from the Fox Chase Cancer Center (FCCC) Biosample Repository (IRB #11-852). Written informed consent was obtained from each patient in accordance with HIPAA-compliant procedures approved by the FCCC Institutional Review Board (IRB #11-866).

### Tumor and placental tissue samples

Lung tissues were isolated from surgical samples and frozen in RNALater and stored at −80°C by the FCCC Biosample Repository (Supplementary Table S2). Placental tissue samples were collected from the retrieved specimen, just after curettage or delivery, and placed in sterile ice-cold 1xPBS until RNA extraction was performed. Blood and blood clots were washed/removed from placental tissues before RNA extraction.

### RNA extraction, reverse transcription, real time PCR (qPCR) and primer set efficiencies

Total RNA was extracted using the RNeasy^®^ Mini Kit (cat# 74104, Qiagen Ltd), and cDNA synthesis using 1μg of total RNA was performed as previously described. qPCR analysis of NKp44 and NKp44 splice variants was performed using the TaqMan^®^ Gene Expression Master Mix (cat# 4369016, Applied Biosystems). qPCR analysis of Np46 and NKp30 and NKp30 splice variants was performed using the Power SYBR^®^ Green PCR master MIX (cat# 4367659, Applied Biosystems). Each reaction contained 30-60ng of cDNA and was performed as previously described [4, 12]. Expression levels of: total NKp46 and NKp30, NKp44-1, NKp44-2, and NKp44- 3 (target genes) were normalized to β-actin (reference gene). Calculation of gene expression was performed using the 2^−ΔCt^ method. The percentage of NKp44 and NKp30 splice variants from total NKp44 or NKp30 mRNA was performed by normalizing the expression of NKp44 or NKp30 splice variants to NKp44 or NKp30 total expression (respectively, reference gene), adding the 2^−ΔCt^ of each splice variant to a total NKp44 or NKp30 expression and calculating the percentage. NKp44-1^dominant^ profile (NKp44-1 in ≥ 66% of total NKp44 transcripts), NKp44-2/3 profile (NKp44-1 in < 66% of total NKp44 transcripts). NKp30a/b profile (% NKp30a ≥ % NKp30c). NKp30c profile (% NKp30a < % NKp30c). Primer set efficiencies. NKp44-total: Fw-primer: 5′-TGATGCTGGCTTCT TCACTG-′3, Probe: 5′-TCTGGTGGTATCTCCAGCCTC TGCCT-′3, Rev-primer: 5′-AGTCCAGGAGGT CTGTG TGG-′3, (eff%:98.6). NKp44-1: Fw-primer-1/3: 5′-ACCA TCCCTGTCC CTTCACAGCCAC −′3, Probe-1/2/3: 5′-GA CTCCTCGTAGCCAAGAGCCTGGTG-′3, Rev-primer-1: 5′-ACCATATGTCCCCCCACCAG-′3, (eff%:99.8). NK p44-2: Fw-primer-2: 5′-CTTCCTGTCCCTC TGCCT TC-′3, Probe NKp44-1/2/3: 5′-GACTCC TCGTAGC CAAGAGCCTGGTG-′3, Rev-primer-2/3: 5′-GATGC TGCAT GTGCCGATTCCTT-′3, (eff%:102.5). NKp44-3: Fw-primer-1/3: 5′-ACCAT CCCTGTCCCT TCACAGCCAC-′3, Probe-1/2/3: 5′-GACTCCTCGTAGCCAAGAGCCTGGTG-′3, Rev-primer-2/3: 5′-GATGCTGCATGTG CCGATTCCTT-′3, (eff%:99.2). NKp46: Fw-primer: 5-GTGACCACAGCCCACCGAG-3, Rev-primer: 5-CTCAATGTCGCCTGTGACCAG-3, (eff%:104.432%), NKp30 total: Fw-primer: 5-CATGGTCC ATCCAGGATCC-3, Rev-primer: 5-GTTCCATTC CTCACCTCCTTC-3, (eff%:100.205%), NKp30-a: Fw-primer:5-GGTGGTGGAGAAAGAACATC-′3, Rev-primer:5-CTTTCCAGGTCAGACATTTGC-3, (eff%: 98.568%), NKp30-b: Fw-primer: 5-GGTGG TGGAGAAAGAACATC-3, Rev-primer: 5-GAGAGT AGATTTGGCATATTTGC-3, (eff%:98.387%), NKp30-c: Fw-primer: 5-GGTGGTGG AGAAAGAA CATC-3, Rev-primer: 5-CATGTGACAGTGGCATTTGC-3, (eff%: 101.560%), β-actin: Fw-primer: 5-GCATTGTTA CCAACTGGGAC-3, Rev-primer: 5-GGTCTC AAACATGATCTGGG-3, (eff%: 103.251%). Target and reference human gene sequences were taken from the National Center for Biotechnology Information (NCBI, www.ncbi.nlm.nih.gov).

### Statistical analysis

Graphics and statistical analysis were performed using GraphPad/Prism5 or Microsoft Office/Excel software. Incidence of NKp44 mRNA and NKp44/NKp30 splice variants profiles statistics were calculated using the fisher exact test. mRNA expression levels of NKp30 and NKp44 statistics between paired samples was calculated using the paired t-test, two-tail. *, p<0.05; **, p<0.01; ***, p<0.001.

## SUPPLEMENTARY FIGURES AND TABLES







## References

[1] Hudspeth K, Silva-Santos B, Mavilio D (2013). Natural cytotoxicity receptors: broader expression patterns and functions in innate and adaptive immune cells. Frontiers in immunology.

[2] Kruse PH, Matta J, Ugolini S, Vivier E (2014). Natural cytotoxicity receptors and their ligands. Immunology and cell biology.

[3] Huret J, Senon S (2006). Atlas of genetics and cytogenetics in oncology and haematology.

[4] Shemesh A, Brusilovsky M, Hadad U, Teltsh O, Edri A, Rubin E, Campbell KS, Rosental B, Porgador A (2016). Survival in acute myeloid leukemia is associated with NKp44 splice variants. Oncotarget.

[5] Rosental B, Brusilovsky M, Hadad U, Oz D, Appel MY, Afergan F, Yossef R, Rosenberg LA, Aharoni A, Cerwenka A, Campbell KS, Braiman A, Porgador A (2011). Proliferating cell nuclear antigen is a novel inhibitory ligand for the natural cytotoxicity receptor NKp44. Journal of immunology (Baltimore, Md: 1950).

[6] Kopcow HD, Allan DS, Chen X, Rybalov B, Andzelm MM, Ge B, Strominger JL (2005). Human decidual NK cells form immature activating synapses and are not cytotoxic. Proceedings of the National Academy of Sciences of the United States of America.

[7] Marlin R, Duriez M, Berkane N, de Truchis C, Madec Y, Rey-Cuille M, Cummings J, Cannou C, Quillay H, Barré-Sinoussi F (2012). Dynamic shift from CD85j/ILT-2 to NKG2D NK receptor expression pattern on human decidual NK during the first trimester of pregnancy. PLoS One.

[8] Delahaye NF, Rusakiewicz S, Martins I, Ménard C, Roux S, Lyonnet L, Paul P, Sarabi M, Chaput N, Semeraro M (2011). Alternatively spliced NKp30 isoforms affect the prognosis of gastrointestinal stromal tumors. Nature medicine.

[9] Messaoudene M, Fregni G, Enot D, Jacquelot N, Neves E, Germaud N, Garchon HJ, Boukouaci W, Tamouza R, Chanal J (2016). NKp30 isoforms and NKp46 transcripts in metastatic melanoma patients: unique NKp30 pattern in rare melanoma patients with favorable evolution. OncoImmunology.

[10] Mantovani S, Mele D, Oliviero B, Barbarini G, Varchetta S, Mondelli MU (2015). NKp30 isoforms in patients with chronic hepatitis C virus infection. Immunology.

[11] Prada N, Antoni G, Commo F, Rusakiewicz S, Semeraro M, Boufassa F, Lambotte O, Meyer L, Gougeon M, Zitvogel L (2013). Analysis of NKp30/NCR3 isoforms in untreated HIV-1-infected patients from the ANRS SEROCO cohort. Oncoimmunology.

[12] Shemesh A, Tirosh D, Sheiner E, Tirosh NB, Brusilovsky M, Segev R, Rosental B, Porgador A (2015). First Trimester Pregnancy Loss and the Expression of Alternatively Spliced NKp30 Isoforms in Maternal Blood and Placental Tissue. Frontiers in immunology.

[13] Mochizuki M, Maruo T, Matsuo H, Samoto T, Ishihara N (1998). Biology of human trophoblast. International Journal of Gynecology & Obstetrics.

[14] Stuart-Harris R, Caldas C, Pinder S, Pharoah P (2008). Proliferation markers and survival in early breast cancer: a systematic review and meta-analysis of 85 studies in 32,825 patients. The Breast.

[15] Holtan SG, Creedon DJ, Haluska P, Markovic SN (2009). Cancer and pregnancy: parallels in growth, invasion, and immune modulation and implications for cancer therapeutic agents.

[16] Levi I, Amsalem H, Nissan A, Darash-Yahana M, Peretz T, Mandelboim O, Rachmilewitz J (2015). Characterization of tumor infiltrating natural killer cell subset. Oncotarget.

[17] Hanna J, Goldman-Wohl D, Hamani Y, Avraham I, Greenfield C, Natanson-Yaron S, Prus D, Cohen-Daniel L, Arnon TI, Manaster I (2006). Decidual NK cells regulate key developmental processes at the human fetal-maternal interface. Nature medicine.

[18] Cerdeira AS, Rajakumar A, Royle CM, Lo A, Husain Z, Thadhani RI, Sukhatme VP, Karumanchi SA, Kopcow HD (2013). Conversion of peripheral blood NK cells to a decidual NK-like phenotype by a cocktail of defined factors. Journal of immunology (Baltimore, Md: 1950).

[19] Keskin DB, Allan DS, Rybalov B, Andzelm MM, Stern JN, Kopcow HD, Koopman LA, Strominger JL (2007). TGFbeta promotes conversion of CD16+ peripheral blood NK cells into CD16- NK cells with similarities to decidual NK cells. Proceedings of the National Academy of Sciences of the United States of America.

[20] Hasmim M, Messai Y, Ziani L, Thiery J, Bouhris J, Noman MZ, Chouaib S (2015). Critical role of tumor microenvironment in shaping NK cell functions: implication of hypoxic stress. Frontiers in immunology.

[21] Hershkovitz O, Jivov S, Bloushtain N, Zilka A, Landau G, Bar-Ilan A, Lichtenstein RG, Campbell KS, van Kuppevelt TH, Porgador A (2007). Characterization of the recognition of tumor cells by the natural cytotoxicity receptor, NKp44. Biochemistry.

[22] Hershkovitz O, Jarahian M, Zilka A, Bar-Ilan A, Landau G, Jivov S, Tekoah Y, Glicklis R, Gallagher JT, Hoffmann SC, Zer H, Mandelboim O, Watzl C (2008). Altered glycosylation of recombinant NKp30 hampers binding to heparan sulfate: a lesson for the use of recombinant immunoreceptors as an immunological tool. Glycobiology.

[23] Rosental B, Hadad U, Brusilovsky M, Campbell KS, Porgador A (2012). A novel mechanism for cancer cells to evade immune attack by NK cells: The interaction between NKp44 and proliferating cell nuclear antigen. Oncoimmunology.

[24] Rajagopalan S, Long EO (2013). Found: a cellular activating ligand for NKp44. Blood.

[25] Brandt CS, Baratin M, Yi EC, Kennedy J, Gao Z, Fox B, Haldeman B, Ostrander CD, Kaifu T, Chabannon C, Moretta A, West R, Xu W (2009). The B7 family member B7-H6 is a tumor cell ligand for the activating natural killer cell receptor NKp30 in humans. The Journal of experimental medicine.

[26] Siewiera J, Gouilly J, Hocine H, Cartron G, Levy C, Al-Daccak R, Jabrane-Ferrat N (2015). Natural cytotoxicity receptor splice variants orchestrate the distinct functions of human natural killer cell subtypes. Nature communications.

[27] Fraser R, Whitley GSJ, Thilaganathan B, Cartwright JE (2015). Decidual natural killer cells regulate vessel stability: implications for impaired spiral artery remodelling. Journal of reproductive immunology.

[28] Kanzaki H (2016). Uterine Endometrial Function. Springer.

[29] Rosinsky C, Antony PA (2016). A role for pre-mNK cells in tumor progression. Journal for immunotherapy of cancer.

[30] Carrega P, Morandi B, Costa R, Frumento G, Forte G, Altavilla G, Ratto GB, Mingari MC, Moretta L, Ferlazzo G (2008). Natural killer cells infiltrating human nonsmall‐cell lung cancer are enriched in CD56brightCD16− cells and display an impaired capability to kill tumor cells. Cancer.

[31] Mamessier E, Sylvain A, Thibult ML, Houvenaeghel G, Jacquemier J, Castellano R, Goncalves A, Andre P, Romagne F, Thibault G, Viens P, Birnbaum D, Bertucci F (2011). Human breast cancer cells enhance self tolerance by promoting evasion from NK cell antitumor immunity. The Journal of clinical investigation.

[32] Gillard-Bocquet M, Caer C, Cagnard N, Crozet L, Perez M, Fridman WH, Sautès-Fridman C, Cremer I (2013). Lung tumor microenvironment induces specific gene expression signature in intratumoral NK cells. Frontiers in immunology.

[33] Takayama T, Kamada N, Chinen H, Okamoto S, Kitazume MT, Chang J, Matuzaki Y, Suzuki S, Sugita A, Koganei K (2010). Imbalance of NKp44 NKp46− and NKp44− NKp46 natural killer cells in the intestinal mucosa of patients with Crohn's disease. Gastroenterology.

[34] Reeves RK, Rajakumar PA, Evans TI, Connole M, Gillis J, Wong FE, Kuzmichev YV, Carville A, Johnson RP (2011). Gut inflammation and indoleamine deoxygenase inhibit IL-17 production and promote cytotoxic potential in NKp44+ mucosal NK cells during SIV infection. Blood.

[35] Li B, Dewey CN (2011). RSEM: accurate transcript quantification from RNA-Seq data with or without a reference genome. BMC bioinformatics.

[36] Platonova S, Cherfils-Vicini J, Damotte D, Crozet L, Vieillard V, Validire P, Andre P, Dieu-Nosjean MC, Alifano M, Regnard JF, Fridman WH, Sautes-Fridman C, Cremer I (2011). Profound coordinated alterations of intratumoral NK cell phenotype and function in lung carcinoma. Cancer research.

[37] Horton NC, Mathew SO, Mathew PA (2013). Novel interaction between proliferating cell nuclear antigen and HLA I on the surface of tumor cells inhibits NK cell function through NKp44. PloS one.

[38] Fuchs A, Cella M, Kondo T, Colonna M (2005). Paradoxic inhibition of human natural interferon-producing cells by the activating receptor NKp44. Blood.

[39] Bashiri A, Shemesh A, Porgador A, Holcberg G, Kabessa M (2016). New Frontiers in RPL Research and Treatment In: Anonymous Recurrent Pregnancy Loss. Springer.

[40] Candido EB, Silva LM, Carvalho AT, Lamaita RM, Filho RM, Cota BD, da Silva-Filho AL (2013). Immune response evaluation through determination of type 1, type 2, and type 17 patterns in patients with epithelial ovarian cancer. Reproductive sciences (Thousand Oaks, Calif).

[41] De Simone V, Franze E, Ronchetti G, Colantoni A, Fantini M, Di Fusco D, Sica G, Sileri P, MacDonald T, Pallone F (2015). Th17-type cytokines, IL-6 and TNF-α synergistically activate STAT3 and NF-kB to promote colorectal cancer cell growth. Oncogene.

[42] Seruga B, Zhang H, Bernstein LJ, Tannock IF (2008). Cytokines and their relationship to the symptoms and outcome of cancer. Nature Reviews Cancer.

[43] Garzetti GG, Ciavattini A, Goteri G, Tranquilli AL, Muzzioli M, Fabris N, De Nictolis M, Romanini C (1994). Natural killer cell activity in stage I endometrial carcinoma: correlation with nuclear grading, myometrial invasion, and immunoreactivity of proliferating cell nuclear antigen. Gynecologic oncology.

