# Supplementary material for: NKp44 and NKp30 splice variant profiles in decidua and tumor tissues: a comparative viewpoint
Source: Oncotarget. 2016 Sep 27;7(43):70912–23. doi: 10.18632/oncotarget.12292 (PMC5342598; doi:10.18632/oncotarget.12292)
Supplement: Supplementary file 1 [file oncotarget-07-70912-s001.pdf]

NKp44 and NKp30 splice variant profiles in decidua and tumor tissues: a comparative viewpoint

SUPPLEMENTARY FIGURES AND TABLES

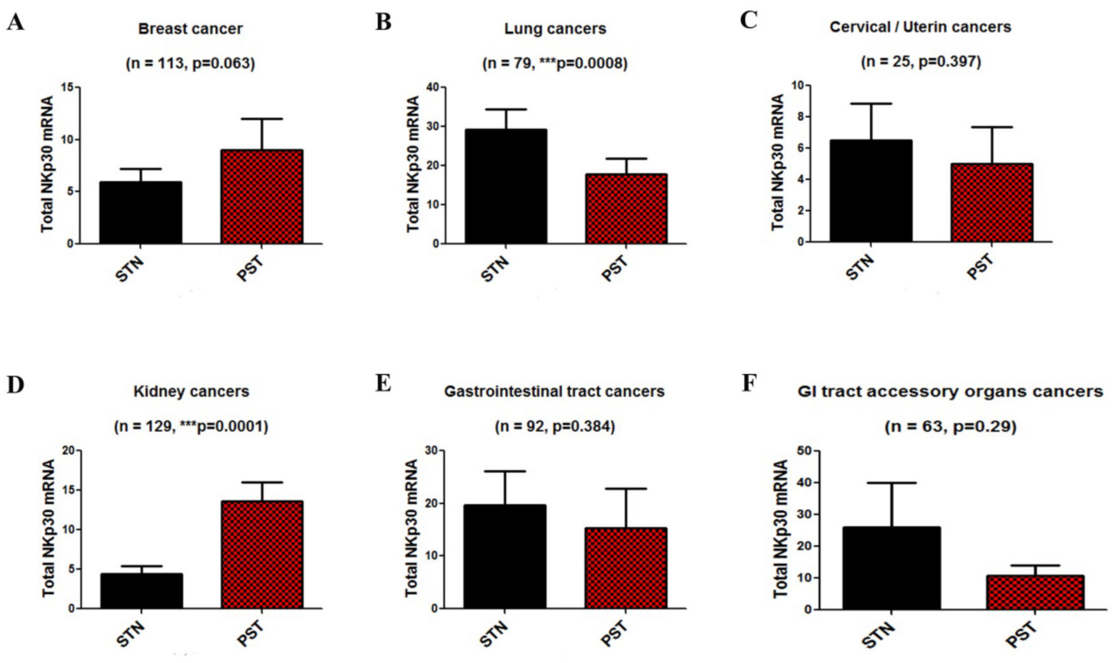

Supplementary Figure S1: NKp30 mRNA expression in cancer cases. RNAseq gene expression data from the TCGA was analyzed for the expression level of NKp30 mRNA in cases of primary solid tumor (PST- red) samples and paired solid tissue normal (STN - black) control samples. Cancer types were clustered by organ. **A.** Breast (BRCA). **B.** Lung (LUAD, LUSC). **C.** Cervical/Uterine (CESC, UCEC). **D.** Kidney (KIRC, KIRP, KIRH) **E.** Gastrointestinal tract organs (ESCA, STAD, COAD, READ) **F.** Gastrointestinal (GI) tract accessory organs (LIHC, PAAD, CHOL). Statistical significance was calculated by paired t-test, two tails. * $p < 0.05$, ** $p < 0.01$. Normalized RNAseq data (RSEM) was obtained from the TCGA (IlluminaHiSeq_RNASeqV2.Level_3).

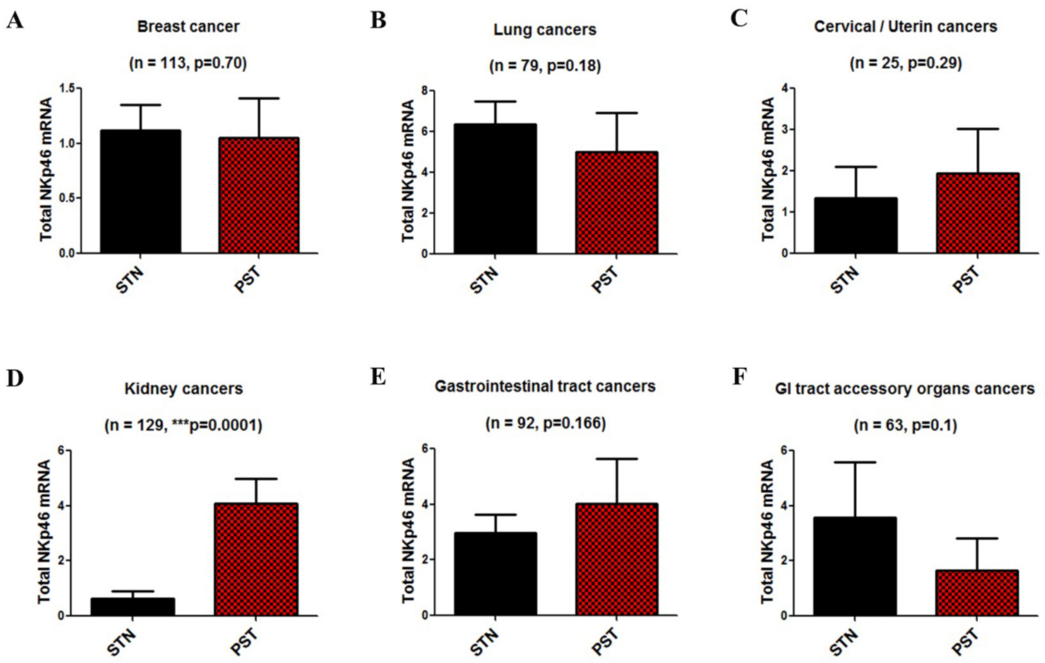

Supplementary Figure S2: NKp46 mRNA expression in cancer cases. RNAseq gene expression data from the TCGA was analyzed for the expression level of NKp46 mRNA in cases of primary solid tumor (PST- red) samples and paired solid tissue normal (STN - black) control samples. Cancer types were clustered by organ. **A.** Breast (BRCA). **B.** Lung (LUAD, LUSC). **C.** Cervical/Uterine (CESC, UCEC). **D.** Kidney (KIRC, KIRP, KIRH) **E.** Gastrointestinal tract organs (ESCA, STAD, COAD, READ) **F.** Gastrointestinal (GI) tract accessory organs (LIHC, PAAD, CHOL). Statistical significance was calculated by paired t-test, two tails. * $p < 0.05$, ** $p < 0.01$. Normalized RNAseq data (RSEM) was obtained from the TCGA (IlluminaHiSeq_RNASeqV2.Level_3).

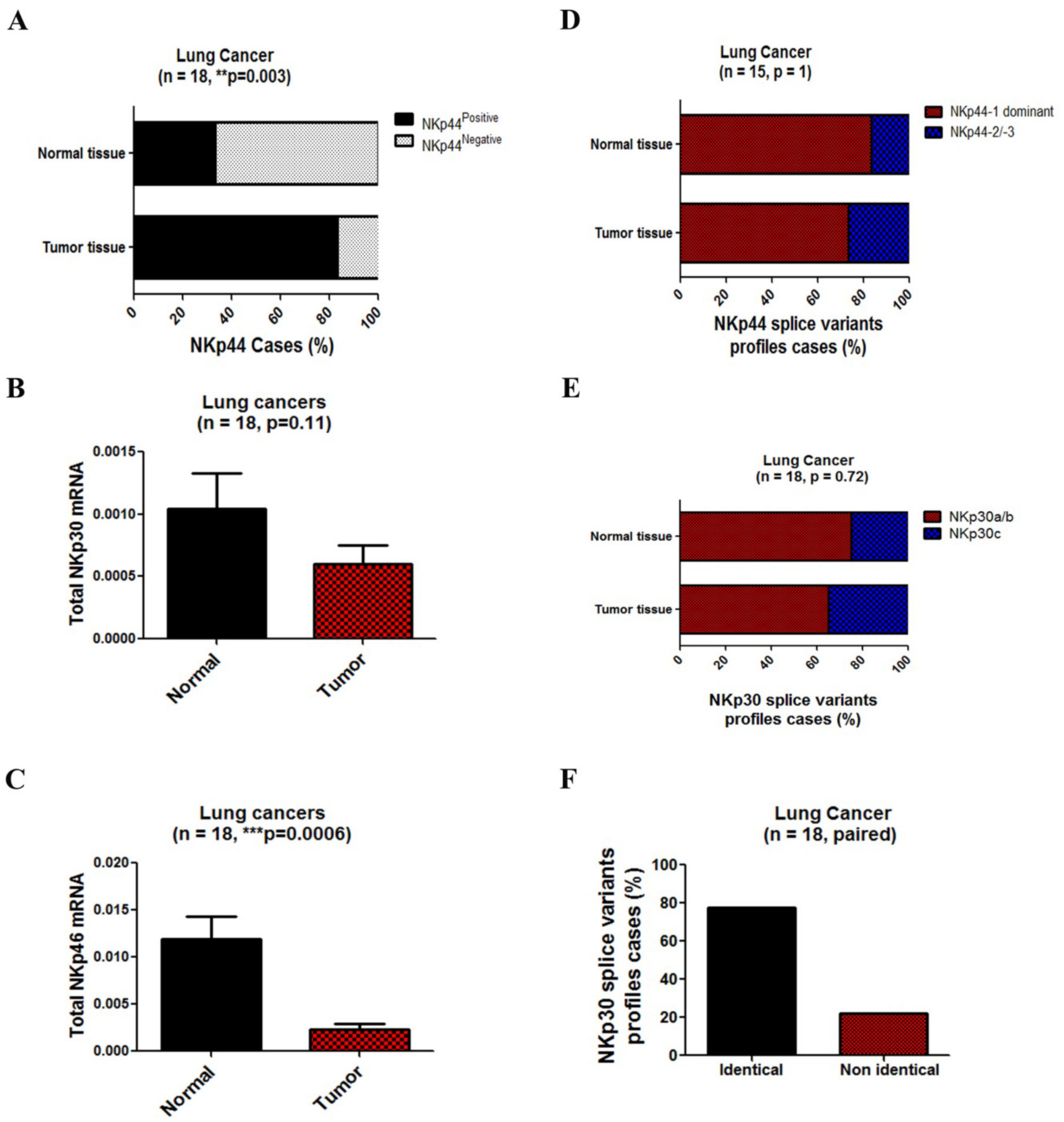

Supplementary Figure S3: Full qPCR analysis of NCRs in human lung cancer samples with matched normal tissue samples. Human Lung tumor samples with matched normal tissue samples were analyzed by qPCR (n = 18). **A.** Percentage of NKp44 positive and NKp44 negative samples in tumor and matched normal tissue samples. Statistical analysis performed using the Fisher exact test, * $p < 0.05$, ** $p < 0.01$. mRNA expression of **B.** NKp30 and **C.** NKp46 in tumor and matched normal tissue samples tissue. Statistical analysis performed using the paired t-test, two-tail, * $p < 0.05$, ** $p < 0.01$, *** $p < 0.001$. **D.** Percentage of NKp44-1^{dominant} profile (NKp44-1 > 66% of NKp44 transcripts – red, inhibitory profile) and NKp44-2/3 profile (NKp44-1 < 66% of NKp44 transcripts – blue, activating profile) in NKp44 positive cases from tumor and normal tissue samples. Statistical analysis performed using the Fisher exact test, * $p < 0.05$, ** $p < 0.01$. **E.** Percentage of NKp30a/b (% NKp30a ≥ % NKp30c – red, activating) and NKp30c (% NKp30a < % NKp30c – blue, inhibitory) in from tumor and normal tissue samples. Fisher exact test, * $p < 0.05$, ** $p < 0.01$. **F.** Percentage of cases that exhibit an NKp30c → a/b (red) or NKp30a/b → c (blue) shift from normal to tumor tissue.

Supplementary Table S1: PCNA expression levels between paired PST and STN samples. RNAseq gene expression data from the TCGA was analyzed for the expression level of PCNA mRNA in cases of primary solid tumor (PST) samples and paired solid tissue normal (STN) control samples. Cancer types were clustered by organ. **i)** Breast (BRCA). **ii)** Lung (LUAD, LUSC). **iii)** Cervical/Uterine (CESC, UCEC). **iv)** Kidney (KIRC, KIRP, KIRH) **v)** Gastrointestinal tract organs (ESCA, STAD, COAD, READ) **vi)** Gastrointestinal (GI) tract accessory organs (LIHC, PAAD, CHOL). Statistical significance was calculated by paired t-test, two tails. Normalized RNAseq data (RSEM) was obtained from the TCGA (IlluminaHiSeq_RNASeqV2.Level_3).

See Supplementary File 1

Supplementary Table S2: FCCC biosample clinical data. Lung cancer patient characteristics (n = 18), Gender: 9 male (50%), 9 female (50%). Race: 16 Caucasian (%), 1 African American (%), 1 Hispanic Caucasian (%). Age at diagnosis: median = 69, range = 60-83. Histology: 9 adenocarcinoma (50%), 8 squamous cell carcinoma (%), 1 papillary squamous (%). Grade: 10 poorly differentiated (%), 6 moderately differentiated (%), 1 well differentiated (%), 1 unknown (%). Tumor NKp44 profile: 11 NKp44-1^{dominant} (61.11%), 4 NKp44-2/3 (22.22%), 3 NKp44 Negative (16.66%). Tumor NKp30 profile: 11 NKp30a/b (61.11%), 7 NKp30c (38.88%).

See Supplementary File 2
